# Prostaglandin E_2_ Production by Brain Endothelial Cells and the Generation of Fever

**DOI:** 10.1089/dna.2022.0662

**Published:** 2023-03-13

**Authors:** Anders Blomqvist

**Affiliations:** Division of Neurobiology, Department of Biomedical and Clinical Sciences, Faculty of Medicine and Health Sciences, Linköping University, Linköping, Sweden.

**Keywords:** cyclooxygenase-2, microsomal prostaglandin E synthase-1, lipopolysaccharide, mice, intravenous, *Slco1c1* promoter

## Abstract

We recently demonstrated that prostaglandin production in brain endothelial cells is both necessary and sufficient for the generation of fever during systemic immune challenge. I here discuss this finding in light of the previous literature and point to some unresolved issues.

Fever is a cardinal symptom of infectious and inflammatory disease, and antipyretic drugs are among the most sold over-the-counter medications in the world. John Vane and his collaborators showed in the early 1970s that the then most commonly used of these drugs, acetylsalicylic acid (aspirin) and paracetamol (acetaminophen), exerted their action by inhibiting prostaglandin synthesis (Flower and Vane, 1972; Vane, 1971).

The prostaglandin synthesizing enzyme was subsequently identified as cyclooxygenase (Cox) and it was shown that aspirin and related drugs act as an active-site acetylating agent for the enzyme (Hemler and Lands, 1976; Roth et al, 1975), whereas paracetamol acts by reducing the active oxidized form of Cox to the resting form (Ouellet and Percival, 2001). The latter finding explains why paracetamol has low effect during conditions with high peroxide load, such as at peripheral inflammatory sites.

With the discovery in 1991 of two isoforms of Cox, a constitutive, Cox-1, and an inducible, Cox-2 (Kujubu et al, 1991; Xie et al, 1991), it became clear that the antipyretic and other anti-inflammatory effects of the cyclooxygenase inhibitors were due to the inhibition of Cox-2, whereas their side effects, such as gastric irritation and bleeding propensity, were caused by the inhibition of Cox-1 (Mitchell et al, 1993).

However, it was not known in which cells the Cox-2, and hence the prostaglandin synthesis that elicited the centrally evoked disease symptoms such as fever, was induced, but in the mid 1990s, two research groups reported induced Cox-2 expression in the rodent brain vasculature after peripheral immune challenge (Breder and Saper, 1996; Cao et al, 1995). However, there was immediately a controversy as to the identity of the vascular cells that has lingered on up to this day. Whereas Saper and his collaborators (Elmquist et al, 1997) suggested that these cells were perivascular macrophages, Watanabe and collaborators identified them as endothelial cells (Cao et al, 1996).

Most subsequent studies, both from the Watanabe laboratory and from other independent research groups, identified, using cell-specific markers, the Cox-2 expression as almost exclusively being in endothelial cells, with few if any Cox-2 expressing cells coexpressing perivascular markers (Chung et al, 2010; Engström et al, 2012; Inoue et al, 2006; Quan et al, 1998; Rivest, 1999).

However, Schiltz and Sawchenko (2002) reported from studies in rats that the perivascular cells exhibited lower threshold to the immune challenge than endothelial cells, an observation that seemed to at least partly offer an explanation for the contradictory data. They reported that intravenous injections of interleukin (IL)-1β and low doses of lipopolysaccharide (LPS) resulted in Cox-2 induction in perivascular macrophages, whereas induction in endothelial cells required higher LPS doses. They suggested that at such higher doses, which generally are used to induce sickness symptoms including fever, the abundant endothelial labeling obscured the sparser perivascular labeling.

The Cox enzymes convert arachidonic acid first to prostaglandin G_2_ (PGG_2_) and then through a peroxidase reaction to prostaglandin H_2_ (PGH_2_). The fate of PGH_2_ is dependent on the presence of terminal isomerases that metabolize this intermediate product into prostacyclin, thromboxane A_2_, prostaglandin D_2_, prostaglandin E_2_, and prostaglandin F_2α_, respectively. The demonstration in 2001 that the brain endothelial cells that expressed Cox-2 upon immune challenge also expressed the equally inducible terminal PGE_2_ isomerase microsomal prostaglandin E synthase (mPGES) (Ek et al, 2001; Yamagata et al, 2001), later renamed mPGES-1, was, therefore, critical in establishing the role for the brain endothelium in PGE_2_ production.

Whereas mPGES-1 is strongly induced in the brain of rats (Damm et al, 2011; Ek et al, 2001; Yamagata et al, 2001), with little constitutive and extravascular expression (Engblom et al, 2002), it is constitutively expressed in the mouse. In this species it is present not only in endothelial cells but also in several other cell types and structures, such as capillary-associated pericytes, astroglial cells, leptomeninges, and the choroid plexus (Eskilsson et al, 2014b). However, colocalization with immune-induced Cox-2 was seen only in the endothelial cells (Eskilsson et al, 2014b), implying that these cells are the site of induced PGE_2_ synthesis in the brain also in mice.

Immune stimulation of mPGES-1 KO mice showed the critical role of this enzyme for the generation of fever. After immune challenge with LPS, such mice showed no fever and no central PGE_2_ synthesis, although they had an intact pyrogenic response to centrally administered PGE_2_ (Engblom et al, 2003). Although these observations, taken together with the demonstration of immune-induced expression of PGE_2_ synthesizing enzymes in the endothelial cells and increased levels of PGE_2_ in the brain (Engblom et al, 2003; Inoue et al, 2002), strongly pointed to these cells as critical for the febrile response, the data showed correlation, not causality.

The first functional evidence for the critical role of brain endothelial cells in fever was given in studies from Ning Quan's and Markus Schwaninger's laboratories, respectively (Ching et al, 2007; Ridder et al, 2011). Ching et al showed impaired fever to IL-1β in a mouse line expressing siRNA against the IL-1 type 1 receptor (IL-1R1) under control of the panendothelial Tie2 promoter. In the Ridder et al study, a mouse line expressing tamoxifen-inducible Cre under the *Slco1c1* promoter, which encodes a thyroxine transporter (Pizzagalli et al, 2002) and which is almost selectively expressed in endothelial cells of the brain and in choroid plexus epithelial cells, was used to delete the MAP kinase kinase kinase TAK1, and it was shown that this procedure attenuated the febrile response to intravenously injected IL-1β.

Subsequently, the same Cre-line was used by us to delete Cox-2 and mPGES-1 in the brain endothelium, similarly resulting in attenuated fever to IL-1β and LPS (Wilhelms et al, 2014), thereby establishing that the PGE_2_ production in the brain endothelial cells was involved in the febrile response. Further studies showed that deletion of the IL-6 receptor α on brain endothelial cells attenuated hypothalamic induction of Cox-2 and the febrile response and that the signaling mechanism involved the STAT3 pathway (Eskilsson et al, 2014a). In a similar way, brain endothelial deletion of IL-1R1, shown to be expressed by the same brain endothelial cells that express Cox-2 and mPGES-1 (Ek et al, 2001; Matsuwaki et al, 2014), attenuated the febrile response (Matsuwaki et al, 2017).

The mentioned data led to the hypothesis that during systemic inflammation, cytokines such as IL-1β and IL-6 are released into the circulation and bind to cognate receptors on brain endothelial cells. This receptor activation in turn elicits intracellular signaling through the NF-*k*B (TAK1) and STAT3 pathways that results in transcriptional upregulation of Cox-2 and mPGES-1 and induced PGE_2_ synthesis.

The thus synthesized PGE_2_ is released in the brain where it through binding to PGE_2_ EP_3_ receptors in the median preoptic nucleus of the hypothalamus activates downstream thermogenic pathways (Lazarus et al, 2007) (Fig. 1). However, it remained unclear if PGE_2_ production in brain endothelial cell was but one among several mechanisms behind fever. During the years other sources of PGE_2_, such as peripheral and central macrophages, as well as prostaglandin-induced neural signaling, have been proposed (see., e.g., Blomqvist and Engblom, 2018).

**FIG. 1.**
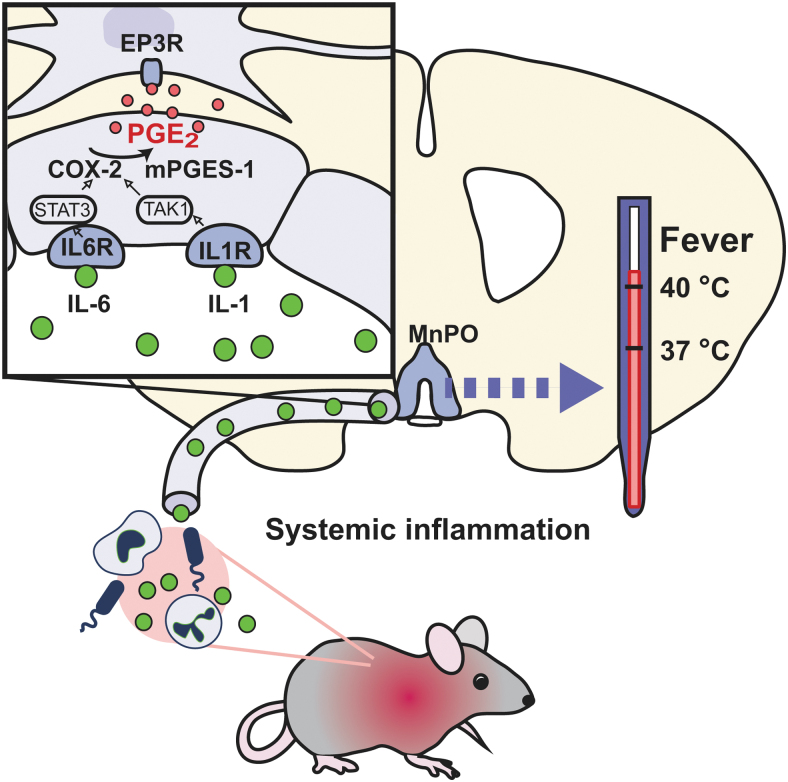
Mechanisms of fever during systemic inflammation. During systemic inflammation, cytokines are released into the circulation. The binding of interleukin (IL)-1 and IL-6 to their receptors (IL1R, IL6R) on brain endothelial cells results, through the TAK1 and STAT3 pathways, in induced expression of cyclooxygenase (Cox)-2 and microsomal prostaglandin E synthase (mPGES)-1. The induction of these enzymes results in the production of PGE_2_ that is released into the brain parenchyma where it by binding to EP_3_ receptors (EP_3_R) in the median preoptic nucleus (MnPO) evokes thermogenesis. Adapted from Eskilsson et al (2021).

In particular, it has been suggested that the initiation of the febrile response is elicited by PGE_2_ production not in the brain but by macrophages in the liver and lungs (Romanovsky et al, 2006; Steiner et al, 2006). In the study using brain endothelial cell deletion of PGE_2_ synthesis (Wilhelms et al, 2014), the immune stimulus was administered through intraperitoneal injection, a procedure that evokes an initial hyperthermia due to the handling stress subjected to the animals and that obscures the initial response to the immunogen (Romanovsky et al, 1998).

We recently addressed these critical issues (Shionoya et al, 2022; Eskilsson et al, 2023). We used a method for delivering the immune stimulus through an indwelling intravenous catheter that did not involve any handling of the animal (Engström et al, 2012; Rudaya et al, 2005), hence permitting detection also of the initial part of the febrile response. By applying this technique to mice with deletion of PGE_2_ synthesizing enzymes in brain endothelial cells, we found that fever was completely abolished when the gene encoding mPGES-1 was deleted (Fig. 2A), whereas an initial febrile response remained when the gene encoding Cox-2 was deleted (Fig. 2B).

**FIG. 2.**
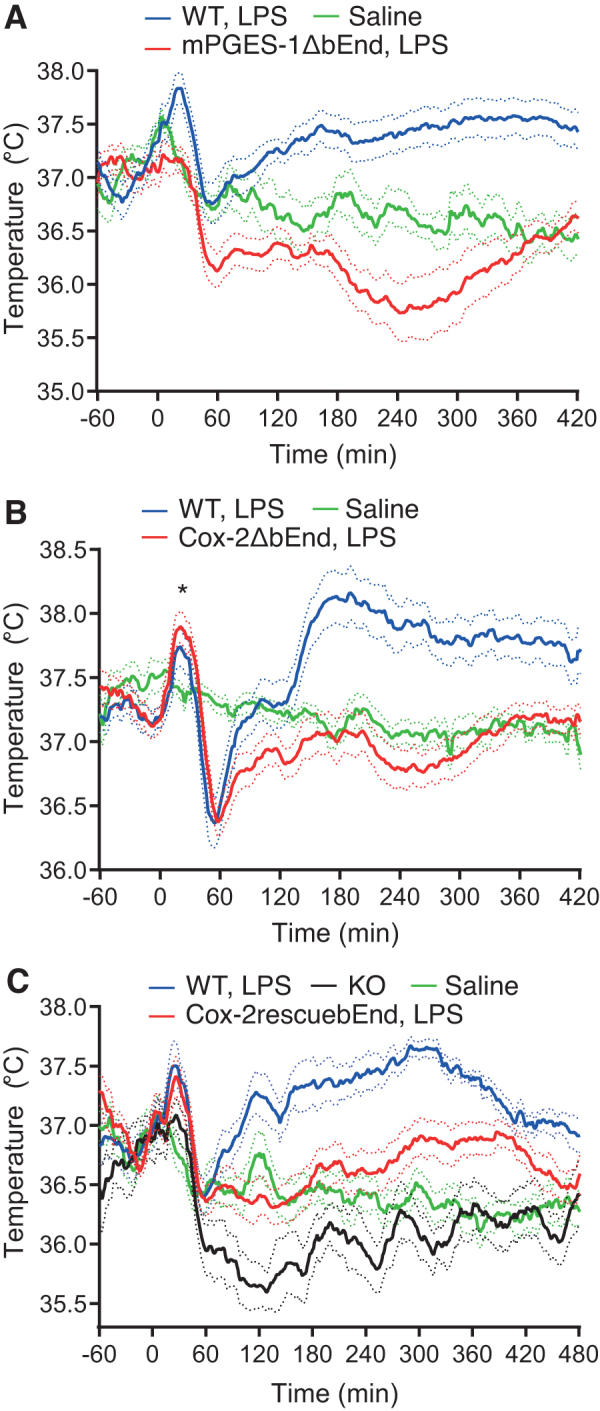
Fever response after brain endothelial cell deletion **(A, B)** and selective expression **(C)** of prostaglandin synthesis. **(A)** Brain endothelial deletion of mPGES-1 (mPGES-1ΔbEnd) abolished the febrile response. **(B)** Brain endothelial deletion of Cox-2 (Cox-2ΔbEnd) abolished the febrile response except for the initial peak (*asterisk*). **(C)** Selective re-expression of Cox-2 in a subset of brain endothelial cells (Cox-2rescuebEnd) resulted in a febrile response. Intravenous injection of LPS was performed at time point 0. *Solid lines* represent mean body temperature and *dotted lines* represent SEM. Adapted from (Shionoya et al, 2022). KO, knock-out; LPS, lipopolysaccharide; WT, wild type.

We interpret this finding that PGE_2_ synthesized in brain endothelial cells is necessary for fever, however, the substrate for mPGES-1, PGH_2_, may during the initial phase of fever also be synthesized by Cox-2 in other cells, such as perivascular macrophages. We then created a mouse line with a *loxP*-flanked transcriptional blocking sequence (loxTB) that prevents normal gene transcription and translation from the endogenous *ptgs2* (Cox-2*)* locus. Selective deletion of the loxTB sequence in brain endothelial cells, that is, permitting Cox-2 expression in these cells only, restored a febrile response to intravenously injected LPS (Fig. 2C). Taken together, the data hence show that prostaglandin production by brain endothelial cells is both necessary and sufficient for the febrile response to systemic inflammation.

Although our findings seem to settle a long-debated issue, there are, as always, several outstanding questions. The model for the generation of fever proposed here seems to be restricted to fever after systemic inflammation, and not for fever evoked by localized inflammation. It was long suggested that such fever was elicited through the activation of peripheral nerves by inflammatory mediators (Miller et al, 1997; Ross et al, 2003; Rummel et al, 2005; Zhang et al, 2008). However, we recently demonstrated that this most likely is not the case (Eskilsson et al, 2021).

We found that whereas global deletion of the PGE_2_ EP_3_ receptor and the IL-1R1 both abolished the febrile response to a localized inflammation (elicited by casein injection into a preformed air pouch), selective deletion of these receptors on peripheral nerves was without effect, implying that activation of peripheral nerves neither by locally produced PGE_2_ nor by IL-1β was responsible for the fever. Furthermore, blockage of the nerves from the inflamed site with a local anesthetic also had no effect on the fever. Surprisingly though, fever also remained after endothelial deletion of Cox-2 or mPGES-1, but was attenuated by endothelial deletion of the IL-1R1 or the IL-6 receptor α.

There was only feeble induction of Cox-2 in the brain, but there were elevated PGE_2_ levels both in the cerebrospinal fluid and in plasma. The findings show that in contrast to what is the case after systemic inflammation, central PGE_2_ production is not essential for the febrile response to peripheral inflammation. Instead, they suggest that the fever during peripheral inflammation is elicited by blood-borne PGE_2_ whose entry into the brain is dependent on cytokine receptor signaling in the brain endothelial cells, the latter known to promote blood–brain barrier permeability (Rochfort and Cummins, 2015).

Another major unresolved question is the function of the immune-induced PGE_2_ production by brain endothelial cells in the parts of the brain not involved in thermoregulation. Induced expression of PGE_2_ synthesizing enzymes is seen throughout the brain, including the cerebral cortex, after peripheral immune challenge (Engblom et al, 2002; Jakobsson et al, 2002). It should in this context be noted that the *Slco1c1-*Cre line we employed for endothelial cell deletions, and which when used to delete mPGES-1 resulted in abolished fever, only recombines in a subpopulation of such cells. It does not recombine in endothelial cells in capillaries, and rarely in large vessels such as those to the neocortical surface and in the meninges, but preferentially in small-to-medium-sized vessels deep in the brain parenchyma (Eskilsson et al, 2017).

Several studies suggest that fever is elicited by the local release, probably in a paracrine manner, of PGE_2_ onto thermoregulatory neurons in the median preoptic nucleus and that it does not depend on the overall PGE_2_ production in the brain (Dinarello and Bernheim, 1982). It was shown that intracerebral injection of a threshold dose of PGE_2_ caused fever when localized to the median preoptic nucleus but not when localized to more distant sites (Scammell et al, 1996), and similarly, microinjections of a cyclooxygenase inhibitor into the same area attenuated LPS-induced fever, whereas microinjections at other sites did not (Scammell et al, 1998).

Along the same vein, we recently showed that local injection of a virus vector expressing mPGES-1 into the median preoptic nucleus of fever-refractive mPGES-1 knockout mice resulted in a temperature elevation in response to LPS (Eskilsson et al, 2017). Although induced prostaglandin production also is involved in other sickness-induced symptoms, such as anorexia and hypothalamic–pituitary–adrenal axis activation (Elander et al, 2009, Elander et al, 2007; Saper et al, 2012), the little evidence that exists points to that these symptoms are not mediated by endothelial prostaglandin production (Ridder et al, 2011).

These data leave most of the PGE_2_ produced in the endothelial cells throughout the brain during inflammation and infection in search for a function. Intriguing hypotheses could be autoregulation of the blood flow (Nippert et al, 2018) and/or involvement in the generalized activation of microglial cells that is seen during peripheral inflammation (Fritz et al, 2016; Klawonn et al, 2021).
